# Myeloid-Derived Suppressor Cells Mediate T Cell Dysfunction in Nonhuman Primate TB Granulomas

**DOI:** 10.1128/mbio.03189-21

**Published:** 2021-12-14

**Authors:** Bindu Singh, Dhiraj K. Singh, Shashank R. Ganatra, Ruby A. Escobedo, Shabaana Khader, Larry S. Schlesinger, Deepak Kaushal, Smriti Mehra

**Affiliations:** a Southwest National Primate Research Center, Texas Biomedical Research Institute, San Antonio, Texas, USA; b Department of Molecular Microbiology, Washington University in St. Louis School of Medicine, St. Louis, Missouri, USA; Texas Christian University

**Keywords:** active TB, CRP, latent TB, myeloid-derived suppressor cells, nonhuman primates, rhesus macaques, bronchoalveolar lavage, peripheral blood mononuclear cell, polymorphonuclear

## Abstract

Myeloid-derived suppressor cells (MDSCs) represent an innate immune cell population comprised of immature myeloid cells and myeloid progenitors with very potent immunosuppressive potential. MDSCs are reported to be abundant in the lungs of active tuberculosis (TB) patients. We sought to perform an in-depth study of MDSCs during latent TB infection (LTBI) and active TB (ATB) using the nonhuman primate (NHP) model of pulmonary TB. We found a higher proportion of granulocytic, polymorphonuclear myeloid-derived suppressor cells (PMN-MDSCs) in the lungs of ATB animals compared to those with LTBI or naive control animals. Active disease in the lung, but not LTBI, was furthermore associated with higher proliferation, expansion, and immunosuppressive capabilities of PMN-MDSCs, as shown by enhanced expression of Ki67, indoleamine 2,3-dioxygenase (IDO1), interleukin-10 (IL-10), matrix metallopeptidase 9 (MMP-9), inducible nitric oxide synthase (iNOS), and programmed death-ligand 1 (PD-L1). These immunosuppressive PMN-MDSCs specifically localized to the lymphocytic cuff at the periphery of the granulomas in animals with ATB. Conversely, these cells were scarcely distributed in interstitial lung tissue and the inner core of granulomas. This spatial regulation suggests an important immunomodulatory role of PMN-MDSCs by restricting T cell access to the TB granuloma core and can potentially explain dysfunctional anti-TB responses in active granuloma. Our results raise the possibility that the presence of MDSCs can serve as a biomarker for ATB, while their disappearance can indicate successful therapy. Furthermore, MDSCs may serve as a potential target cell for adjunctive TB therapy.

## INTRODUCTION

Tuberculosis (TB) was the deadliest infectious disease of humanity prior to the onset of the COVID-19 pandemic. In the past year, COVID-19 became a global health priority, but with the availability of several efficacious vaccines against COVID-19, TB is widely expected to regain its place as the infectious disease leading to the greatest mortality worldwide (1). The vast majority of individuals can control infection with Mycobacterium tuberculosis, the causative organism of TB, by restricting the pathogen and associated immune response to localized tissue environments called granulomas. This is believed to be the basis of latency in M. tuberculosis infection (2, 3). Yet, in a number of cases, latent TB infection (LTBI) either is not established or recrudescence can occur after the initial establishment of latency. Having coevolved with its human host, M. tuberculosis has developed multiple host immune evasion mechanisms during the course of its infection. In fact, the development of active disease is chiefly driven by dysregulation of effective immunity (4).

### Classification and characterization of MDSCs.

M. tuberculosis primarily infects macrophages, and cells of myeloid origin mount the initial response to infection (5). Recent studies have established myeloid-derived suppressor cells (MDSCs) as an innate immune population comprised of immature myeloid cells and myeloid progenitors with very potent immunosuppressive potential. MDSCs represent a unique population of myeloid cells that deviate from their regular fate to differentiate into macrophages, granulocytes, or dendritic cells (DCs) under physiological conditions like aging (6) or pathological conditions like chronic inflammation (7–11) or cancer (12–15). They are usually classified into monocytic MDSCs (M-MDSCs) or polymorphonuclear MDSCs (PMN-MDSCs) based on their nuclear organization and expression of key surface receptors. In humans, both of these subsets express CD11b. M-MDSCs are mononuclear and express CD14, while PMN-MDSCs exhibit a polymorphic nuclear organization with the expression of CD15 and CD66b. Both the MDSC subsets express the general myeloid marker CD33 in humans and lack lineage markers for lymphocytes and NK cells. While the M-MDSC markers overlap with monocytes, M-MDSCs are distinguished from monocytes by their absence of HLA-DR expression. MDSCs develop from bone marrow hematopoietic precursors due to altered myelopoiesis induced by sustained inflammatory response, although their development from mature myeloid cell populations cannot be ruled out yet.

In human blood, M-MDSCs are present in the same density fraction as monocytes, but differ from the latter by the virtual absence of HLA-DR expression. They can accurately be characterized as lymphocyte lineage marker-negative cells with a CD11b^+^ or CD33^+^/HLA-DR^−^ CD14^+^ CD15^−^ phenotype. M-MDSCs display high CD33 expression compared to PMN-MDSCs. Murine MDSCs express Gr1 and CD11b, along with variable expression of Ly6C and Ly6G. The two major subsets are characterized as Gr-1^+^ CD11b^+^ Ly6G^−/lo^ Ly6C^hi^ for M-MDSCs and CD11b^+^ Ly6G^+^ Ly6C^lo/int^ for PMN-MDSCs (16). PMN-MDSCs express other markers, including activation markers like CD62L, CD54/ICAM-1, CD63, and CD274/PD-L1, chemokine receptors like CXCR2 and CXCR4, and functional markers like arginase 1 (ARG1), lectin-like oxidized low-density lipoprotein (LDL) receptor-1 (Lox-1), and indoleamine 2,3-dioxygenase (IDO1).

### MDSCs—potent suppressors of immune functions.

MDSCs have been well studied in neoplastic malignancies and conclusively shown to induce their immunosuppressive effects on antigen-specific T cells, spanning both the priming as well as effector phases of tumor-infiltrating lymphocytes (TILs) (17). MDSCs also inhibit effector responses like cytokine responses, proliferation, and lytic activities of TILs (18). The loss of these functions is not due to exhaustion, as purification and antigenic challenges post-*in vitro* culture have been reported to restore the effector responses in TILs (18–21). Microscopic colocalization interrogations in primary human cancer tissues have established the MDSC-T cell immunological synapses to reside within the intratumoral regions (22). The magnitude of suppressive effects of MDSCs on TILs has a direct correlation with proximal localization and physical engagement (22). Apart from cancer, transplantation, and inflammatory pathologies, where these cells are extensively studied (12–15), MDSCs have been recently reported to be abundant in the blood (7, 23, 24), pleural effusions (7), and bronchoalveolar lavage (BAL) fluid (23) of active TB (ATB) patients. MDSCs could potently impair adaptive immunity by killing DCs (25) or inhibiting T cell functions (7, 9, 10, 22, 25–35).

MDSC-driven T cell dysfunction spans inhibition of activation, proliferation, and/or effector functions of both CD4^+^ helper T cells and CD8^+^ cytotoxic T cells by multiple mechanisms. These include metabolite-driven suppression via IDO1, inducible nitric oxide synthase (iNOS), etc., or initiation of an anti-inflammatory cytokine cascade driven by interleukin-10 (IL-10), transforming growth factor β (TGF-β), etc., or via direct cell contact-dependent inhibition mediated by transfer of cytoplasmic material to T cells from MDSCs (10, 25, 29–36). Myelopoiesis-promoting growth factors, such as granulocyte colony-stimulating factor (G-CSF) (37, 38), macrophage CSF (M-CSF), and granulocyte-macrophage CSF (GMCSF) (39), act together with inflammatory cytokines to promote MDSC expansion and to prevent their differentiation into terminally differentiated DCs, macrophages, or granulocytes (39). MDSCs potently suppress the function of both CD4 and CD8 T cells (10, 22, 25–33, 35, 36). Adoptive transferred MDSCs can induce antigen-specific CD8 T cell tolerance in mice (40). Compared with other tolerogenic myeloid cells (e.g., regulatory DCs or regulatory macrophages) (12–15), MDSCs are unique in that they can be activated to suppress immunity by inflammatory signals (7, 8, 11, 25, 30, 32). These include proinflammatory cytokines that are elevated after surgery and Toll-like receptor ligands, making MDSCs potentially well suited for targeting by adjunctive immunotherapeutics (11, 25, 30, 32, 34, 35, 41). MDSCs have recently received attention as potential targets for anti-TB immunotherapy (11, 25, 30, 32, 34, 35, 41).

### MDSCs in tuberculosis.

Although there exist limited data on the role of MDSCs in TB, several studies in murine models of TB, particularly C3HeB/FeJ, C57BL/6, 129S2, NOS2^−/−^, and RAG2^−/−^mice have shown that MDSCs are generated during TB infection. The immunocompromised mice possessed higher frequencies of MDSCs compared to TB-resistant or wild-type mice, where these cells lead to suppression of T cell proliferation and functions. With the course of TB progression, the accumulation of MDSCs is accompanied by the loss of conventional neutrophils. Selective depletion of MDSCs in C3HeB/FeJ mice has been shown to reduce bacillary burden and promote T helper 1 (TH1) responses (42). The presence of a high number of MDSCs correlates with increased surface expression of IL-4 receptor α (IL-4Rα), resulting in an inability to control M. tuberculosis infection, thereby leading to profound TB progression and lethality (9, 43).

In humans, the frequency and effect of MDSCs have been studied in the blood of ATB and LTBI patients. Blood from ATB individuals was reported to possess significantly higher MDSC frequencies than blood from individuals with LTBI. Moreover, MDSCs isolated from ATB patients were able to suppress T cell proliferations by up to 72% and also subvert the mycobacterial containment (44). The findings that MDSCs provide a niche for M. tuberculosis survival, which in turn results in immunity to TB, advocates that MDSCs can serve as susceptible targets for host-directed therapies for TB prevention (45).

### Characterization of MDSCs in NHP model of TB.

Nonhuman primates (NHPs) are an important animal model of TB (46) and, in particular, a model of M. tuberculosis-host interaction within human-like granulomas, and as such are ideal for testing anti-TB immunotherapeutics (47). MDSCs have been well characterized in mice and humans (48–50); however, only limited information is available about these cells in NHPs (39), particularly in the NHP TB model. Recently, Sui et al. demonstrated an increase in MDSCs in TB-vaccinated rhesus macaques (RMs) and the ability of these cells to suppress T cell responses *in vitro* (26). Due to variations in immunocyte phenotypes among humans and RMs, along with limited availability and repertoire of cross-reactive antibodies, phenotypic approaches used for characterizing human MDSCs cannot be directly employed for characterizing rhesus MDSCs. A major phenotypic difference among immune cells of humans and RMs is differential expression of CD56, which is a unique lineage-determining marker for human NK cells and is also present on a subset of monocytes in RMs. Alternatively, CD8/NKG2A expressed on NK cells in RMs can be used along with CD3 and CD20 to exclude lymphoid cells in a gating strategy to identify rhesus MDSCs. CD14 expression can be used to identify monocytes and M-MDSCs in both RMs and humans (51, 52). Similarly, CD66abce can be used to characterize neutrophils and PMN-MDSCs in both humans and RMs (51, 52). CD33 is a key marker used to identify human MDSCs, with high expression on human M-MDSCs and intermediate expression on PMN-MDSCs. However, only one antibody clone has been validated to recognize CD33 in RMs (53). In RMs, immunosuppressive functions have been attributed to a monocyte subpopulation lacking HLA-DR expression (51). The RM low-density neutrophil (LDN) population comprises both CD33^−^ and CD33^+^ cells, of which only the CD33^+^ subset can exert immunosuppressive functions by inhibiting T cell responses and is thus defined as PMN-MDSCs (53).

We sought to conduct an in-depth characterization of MDSCs during latent TB and ATB by using the NHP model of pulmonary TB at the site of infection. We first used high-parameter flow cytometry to develop gating strategies to identify different types of MDSCs (PMN- versus M-MDSCs) in the lung compartment in M. tuberculosis-infected rhesus macaques (RMs). Next, we employed multicolor confocal imaging in lung sections to phenotype MDSCs and elucidate their spatial localization with respect to TB granuloma in NHP lungs. Our aim was to define the spatial distribution of MDSCs in lung TB granulomas and gain better insight into their potential role in driving the granulomatous architecture. Our study demonstrates that ATB is characterized by a measurable increase in MDSC levels in both peripheral blood and BAL fluid, with significant recruitment of these cells to the outer periphery of the granulomas. Our results raise the possibility that MDSCs can serve as a promising biomarker for ATB, while their disappearance could correlate with successful therapy (7–9, 11, 30, 33). Furthermore, they may serve as a potential target for adjunctive TB therapeutics (9, 11, 25, 30, 32, 35, 36, 41), helping to resolve the granulomatous TB niche and consequently the potential for reactivation from latent TB to ATB.

## RESULTS

### Gating strategy for MDSC characterization in RMs.

For defining MDSCs in RM for this study, we used the gating strategy depicted in Fig. S1A in the supplemental material. Briefly, M-MDSCs were gated as CD3^−^ CD20^−^ CD66abce^−^ CD14^+^ HLA-DR^−^ and PMN-MDSCs gated as CD3^−^ CD20^−^ CD66abce^+^ CD14^−/INT^ HLA-DR^−^ CD33^+^. The comparative analysis of phenotypic differences among gated PMN-MDSCs and M-MDSCs is depicted in Fig. S1B.

10.1128/mbio.03189-21.1 FIG S1 (A) Gating strategy used to estimate MDSCs in NHP lungs. (B) Comparative analysis of phenotypic differences among gated PMN-MDSCs and M-MDSCs. Download FIG S1, PDF file, 0.8 MB. Copyright © 2021 Singh et al. 2021 Singh et al. https://creativecommons.org/licenses/by/4.0/ This content is distributed under the terms of the Creative Commons Attribution 4.0 International license.

### Distribution of MDSCs in ATB, LTBI, and naive RMs.

**FIG 1:**
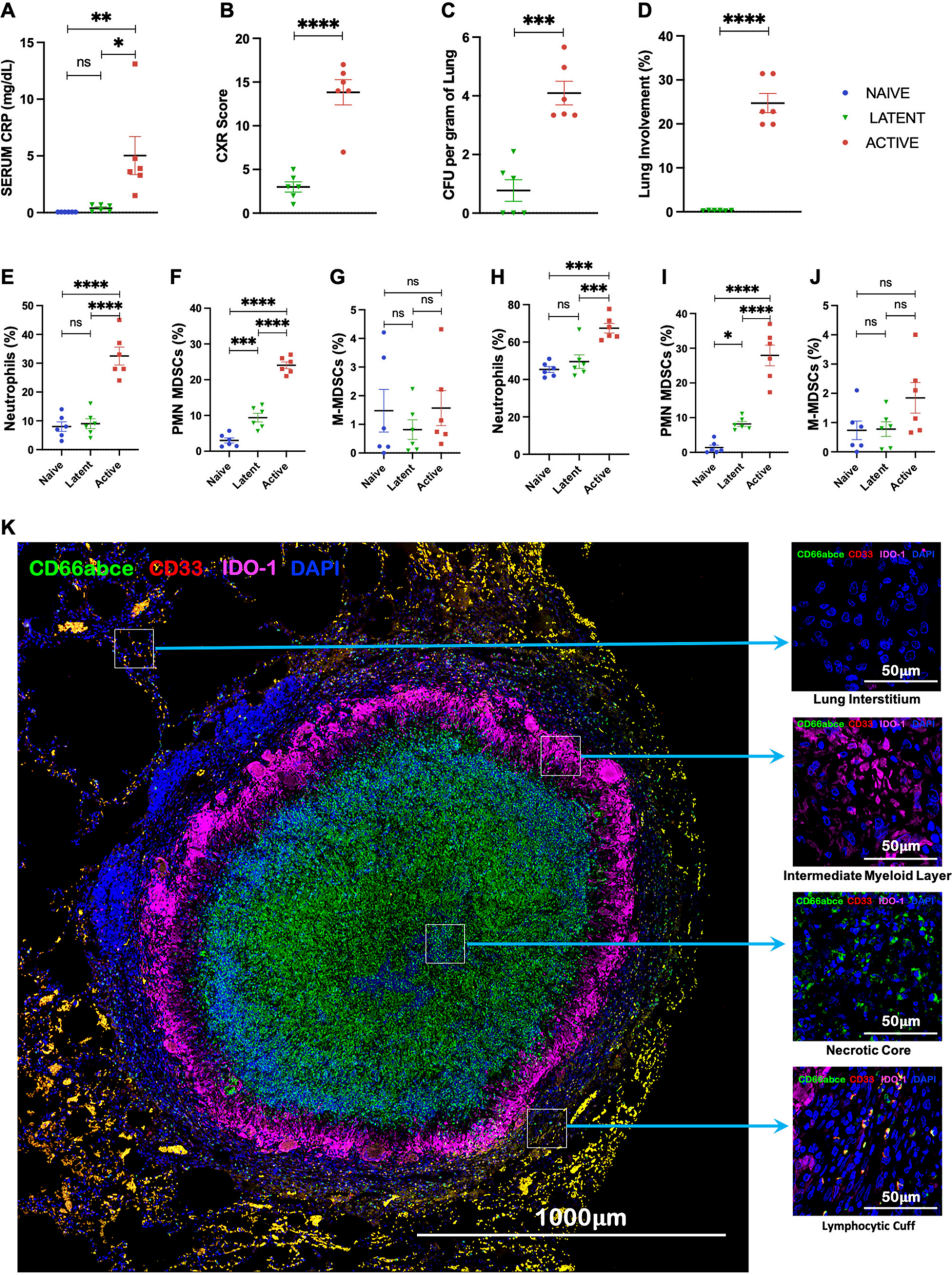
MDSCs in peripheral blood correlate with disease severity in M. tuberculosis-infected RMs. Shown is a graphical representation of correlates of TB disease in M. tuberculosis-infected macaques showing severity of disease in ATB (red) and LTBI (green). (A) Serum CRP level. (B) CXR score. (C) Bacterial burden at necropsy measured in lung (CFU/g of lung). (D) Lung involvement, quantified for every animal as the percentage of the lung with histopathologic abnormalities, including granulomatous inflammation, necrosis, hemorrhage, and edema. (E to J) Comparison of neutrophils, PMNs, and M-MDSCs in the three study groups: ATB (red), LTBI (green), and naive control (blue). (E and H) Percentage of neutrophils (PMNs) in BAL fluid (E) and blood (H). (F and I) Fractions of PMN-MDSCs gated as CD3^−^ CD20^−^ CD66abce^+^ CD14^−/int^ HLA-DR^−^ CD33^+^ in BAL fluid (F) and peripheral blood (I) at necropsy. (G and J) Fraction of M-MDSCs gated as CD3^−^ CD20^−^ CD66abce^−^ CD14^+^ HLA-DR^−^ in BAL fluid (G) and blood (J). (K) Multilabel immunofluorescence tiled image of a TB granuloma from an M. tuberculosis-infected lung showing IDO1 expression in PMN-MDSCs (marked by CD66abce and CD33). (B to D) Statistical significance by two-tailed unpaired *t* test with no correction: *, *P* < 0.05; **, *P* < 0.01; ***, *P* < 0.001; ****, *P* < 0.0001. (A and E to J) Statistical significance by one-way ANOVA with Tukey’s multiple-testing correction: *, *P* < 0.05; **, *P* < 0.01; ***, *P* < 0.001; ****, *P* < 0.0001. Data represent mean ± standard error of the mean (SEM). Naive mice (*n* = 6) are shown in blue, M. tuberculosis CDC1551-infected LTBI mice (*n* = 6) are shown in green, and M. tuberculosis CDC1551-infected ATB mice (*n* = 6) are shown in red.

To study the role of MDSCs during ATB and LTBI, we used the NHP model of pulmonary TB. We randomly selected six TB-naive RMs, six with asymptomatic LTBI after 9 to 12 weeks of exposure to a low dose of M. tuberculosis, and an equal number that developed active TB after exposure to a high dose of M. tuberculosis, as shown by serum C-reactive protein (CRP) (Fig. 1A), chest X-ray (CXR) score (Fig. 1B), lung M. tuberculosis burden (Fig. 1C), and pathology (Fig. 1D). Across different groups, RMs displayed significant changes in the distribution of neutrophils (Fig. 1E and H) and PMN-MDSCs (Fig. 1F and I) in both BAL fluid and peripheral blood, respectively. These results correlated directly with lung levels of M. tuberculosis at the endpoint, disease severity, and biochemistry and blood profile representative of ATB and LTBI (see Fig. S2 in the supplemental material). While no significant differences in M-MDSC abundance were observed in BAL fluid (Fig. 1G) and peripheral blood (Fig. 1J), a subset of NHPs recorded higher M-MDSC fractions in peripheral blood of ATB animals (Fig. 1J).

10.1128/mbio.03189-21.2 FIG S2 Peripheral blood complete blood counts and biochemistry (CBC/chem) in the three study groups: ATB (red), LTBI (green), and naive control (blue). (A) PMN/lymphocyte ratio (NLR), (B) lymphocyte/monocyte ratio (LMR), (C) serum albumin/globulin ratio (AGR), (D) serum albumin level, and (E) serum globulin level were estimated for each animal at necropsy. Statistical significance by one-way ANOVA with Tukey’s multiple-testing correction: **, *P* < 0.01; ***, *P* < 0.001; ****, *P* < 0.0001. Data represent mean ± SEM. Download FIG S2, TIFF file, 1.3 MB. Copyright © 2021 Singh et al. 2021 Singh et al. https://creativecommons.org/licenses/by/4.0/ This content is distributed under the terms of the Creative Commons Attribution 4.0 International license.

### Abundance of MDSCs among the three study groups—ATB, LTBI, and naive RMs.

**FIG 2:**
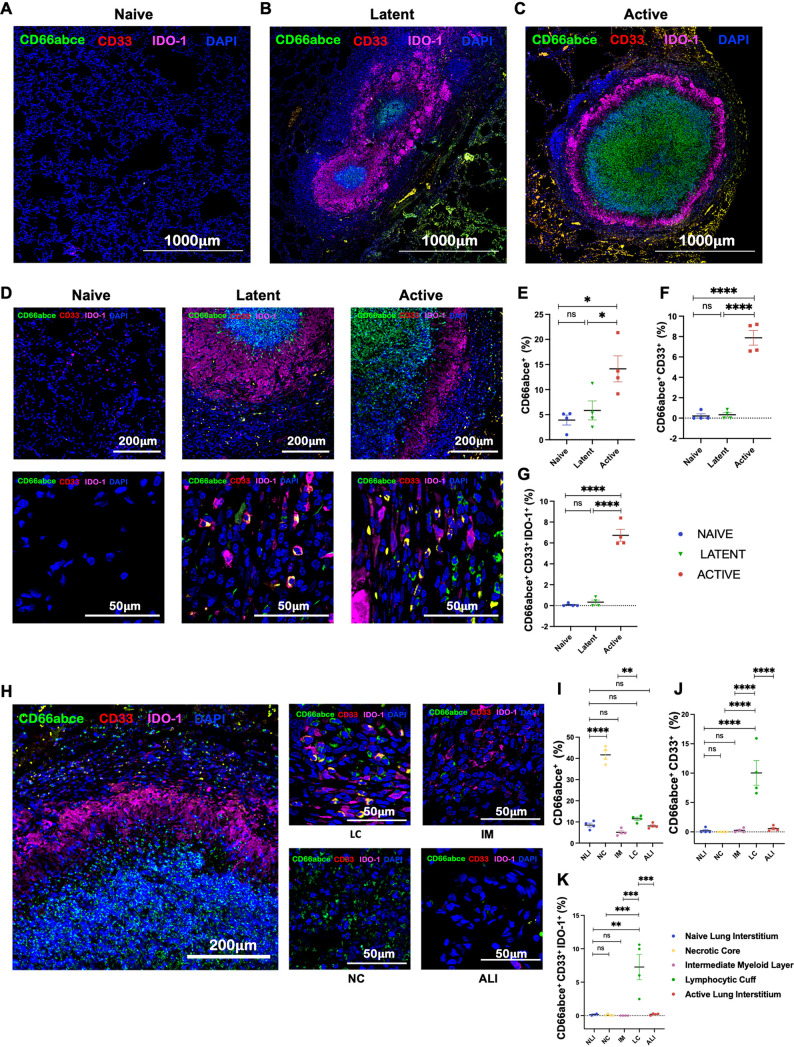
MDSCs induced in TB localize to the lymphocytic cuff at the periphery of granuloma. Comparison of PMN-MDSCs in lungs of the three study groups: ATB (red), LTBI (green), and naive control (blue). Shown are the stitched confocal images (4-by-4 fields at ×10 magnification) of formalin-fixed and paraffin-embedded lung sections depicting control naive lung (A) and granuloma obtained from LTBI (B) and ATB RMs (C). Images from naive, LTBI, and ATB lung depicting PMN-MDSCs stained with CD66abce (green), CD33 (red), IDO1 (magenta), and DAPI (blue) at ×10 and ×63 magnification (D). Estimated fractions of PMNs (CD66abce^+^) (E), PMN-MDSCs (CD66abce^+^ CD33^+^) (F), and IDO1^+^ PMN-MDSCs (CD66abce^+^ CD33^+^ IDO1^+^) (G) are shown in lung sections of ATB (red), LTBI (green), and naive (blue) macaques. (H) Images showing the distribution of PMN-MDSCs in various regions of granuloma and active lung interstitium. (I to K) Graphs showing estimated fractions of PMNs (CD66abce^+^) (I), PMN-MDSCs (CD66abce^+^ CD33^+^) (J), and PMN-MDSCs (CD66abce^+^ CD33^+^ IDO1^+^) (K) in different regions of granuloma: the necrotic core (NC), intermediate myeloid layer (IM), lymphocytic cuff (LC), active TB lung interstitium (ALI), and naive lung interstitium (NLI). Statistical significance by one-way ANOVA with Tukey’s multiple-testing correction: *, *P* < 0.05; **, *P* < 0.01; ***, *P* < 0.001; ****, *P* < 0.0001. Data represent mean ± SEM (*n* = 4).

To further understand the localization of MDSCs at the primary site of infection (i.e., the lungs), we stained formalin-fixed and paraffin-embedded lung sections from naive, LTBI, and ATB animals with markers specific for MDSCs, as well as other functional markers, and analyzed the results using confocal microscopy. The sections were stained for PMNs as well as M-MDSCs using multilabel fluorescence immunohistochemistry. PMN-MDSCs were identified as the granulocytes coexpressing CD66abce (a marker for PMNs in RM and in humans) and CD33 (characteristic surface marker for MDSCs). Furthermore, PMN-MDSCs (CD66abce^+^ CD33^+^) were stained for several other markers that correlate with the immunosuppressive nature of MDSCs. M-MDSCs were identified as mononuclear cells expressing CD14 but not HLA-DR (CD14^+^ HLA-DR^−^). Macaques form human-like granulomas during M. tuberculosis infection (46), which are highly organized and structured with variable cellular composition across different layers (Fig. 1K). The core is usually comprised of caseum rich in neutrophils, surrounded by a layer of myeloid cells—mainly macrophages and giant cells—which are surrounded by a lymphocyte-rich cuff at the periphery of the granuloma (Fig. 1K). We assessed differences in MDSC abundance in the lungs of ATB, LTBI, and naive NHPs and also assessed the spatial localization of MDSCs in different layers of granuloma. We observed an increase in frequencies of lung neutrophils and PMN-MDSC populations that correlated with the severity of disease (Fig. 2A to F). Furthermore, PMN-MDSCs from animals with active TB demonstrated a higher immunosuppressive potential, as indicated by higher expression of IDO1 (Fig. 2A to G). The core of the granuloma was comprised of mainly neutrophils and was almost completely devoid of PMN-MDSCs (Fig. 2H to J). The intermediate zone of granuloma is comprised mostly of myeloid cells and is demarcated by high IDO1 expression. PMN-MDSCs were only very sparsely present in this zone (Fig. 2H to J). PMN-MDSCs were also scarce in the interstitial lung tissue of ATB animals (Fig. 2J). Throughout all lung sections, PMN-MDSCs were almost completely localized to the lymphocytic cuff at the periphery of the granuloma (Fig. 2H to J). PMN-MDSCs localizing to the lymphocytic cuff also had a higher expression of IDO1 (Fig. 2H and K).

### Expression of immunosuppressive effectors/mediators on RM MDSCs.

**FIG 3:**
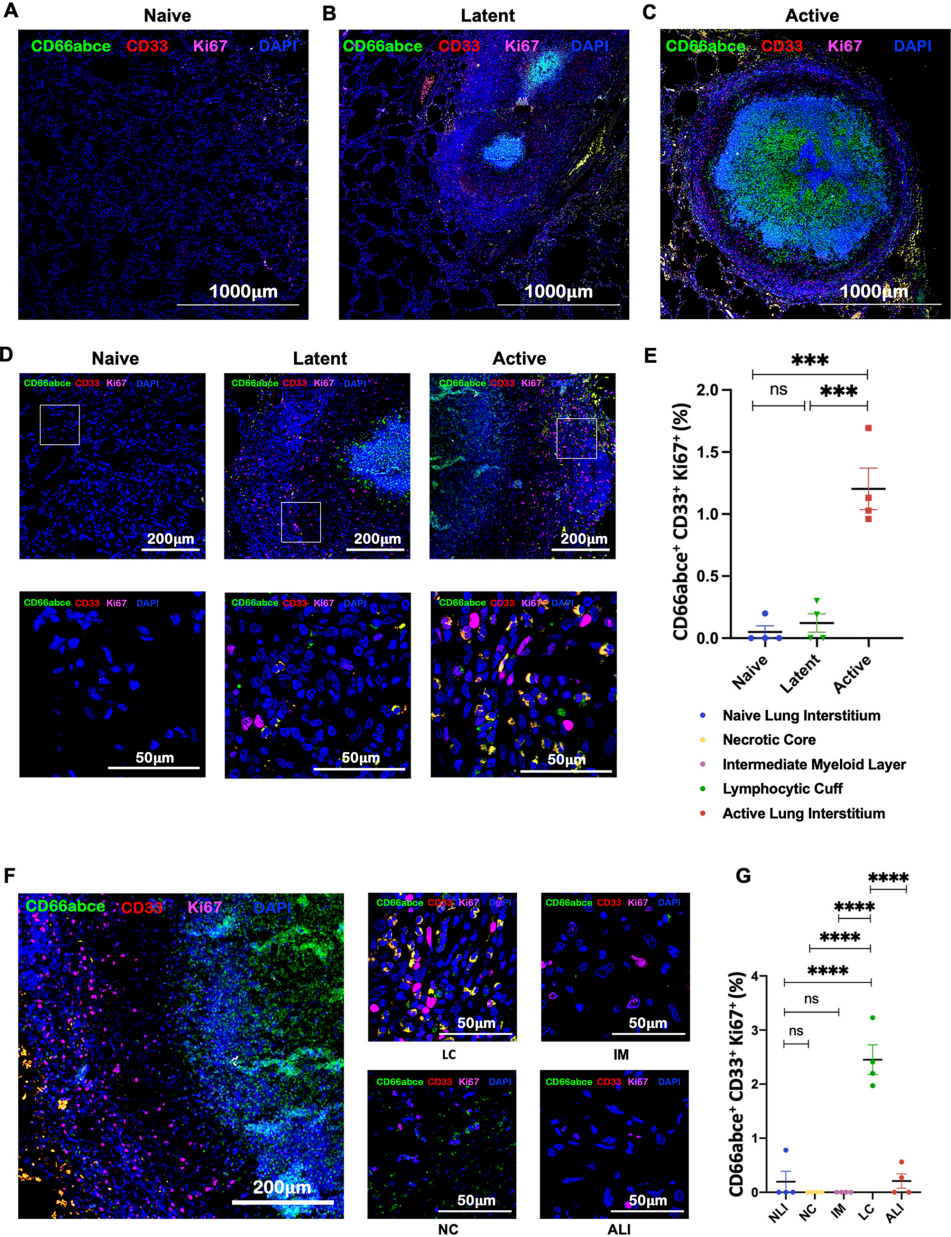
Highly proliferative phenotype in MDSCs recruited to the lungs of RMs with ATB. Lung sections from ATB, LTBI, and naive groups were stained for CD66abce, CD33, and Ki67. Shown are the stitched confocal images (4-by-4 fields at ×10) of formalin-fixed and paraffin-embedded lung sections depicting proliferating PMN-MDSCs in control naive lung (A) and granuloma obtained from LTBI (B) and RMs with ATB (C). (D) Multilabel immunohistochemistry images exhibiting the distribution of proliferating PMN-MDSCs stained with CD66abce (green), CD33 (red), Ki67 (magenta), and DAPI (blue) in naive, LTBI, and ATB lung sections. (E) Graphical representation depicting a comparison of estimated fractions of Ki67^+^ PMN-MDSCs among the three groups. (F) Confocal images showing the distribution of proliferating PMN-MDSCs in various regions of a granuloma in ATB lung. (G) Estimated fraction of proliferating PMN-MDSCs (CD66abce^+^ CD33^+^ Ki67^+^) in the necrotic core (NC), intermediate myeloid layer (IM), lymphocytic cuff (LC), active lung interstitium (ALI), and naive lung interstitium (NLI). Statistical significance by one-way ANOVA with Tukey’s multiple-testing correction: *, *P* < 0.05; ****, *P* < 0.0001. Data represent mean ± SEM (*n* = 4).

**FIG 4:**
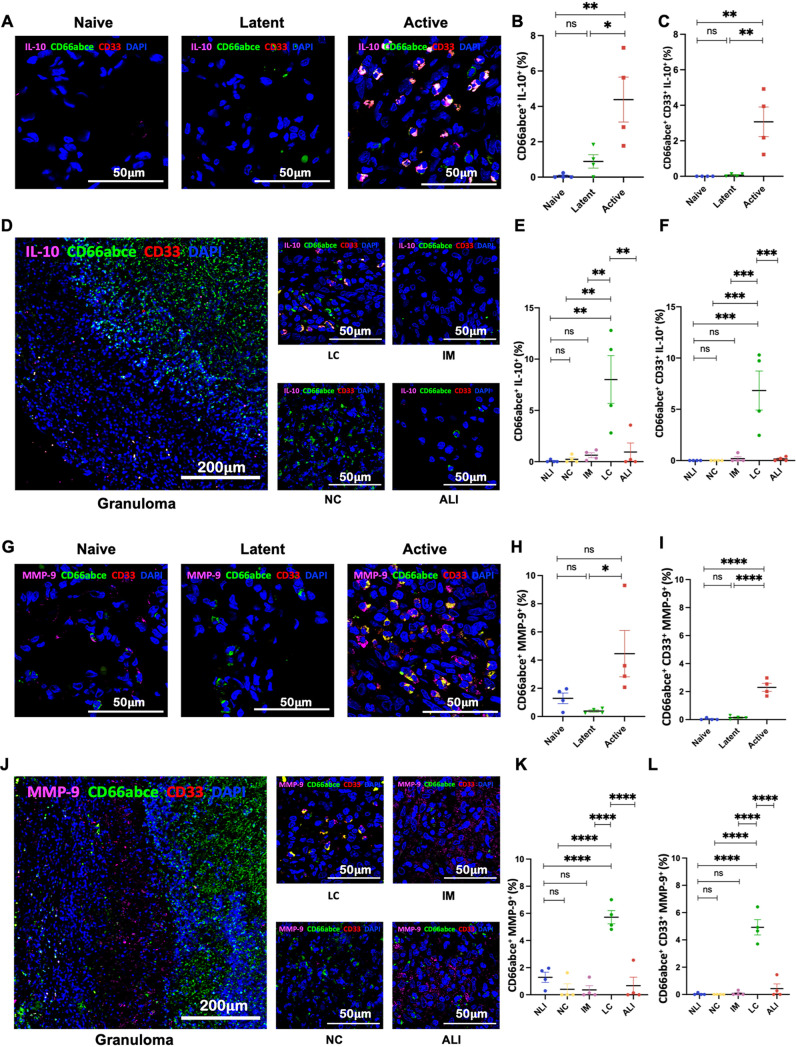
PMN-MDSCs recruited to the lungs of M. tuberculosis-infected macaques express immunomodulatory cytokines and matrix metallopeptidase. (A) Confocal images depicting the expression of immunomodulatory molecule IL-10 by PMN-MDSCs in lungs of naive, LTBI, and ATB lung sections. (B and C) Graphs showing the fractions of IL-10^+^ PMNs (CD66abce^+^ IL-10^+^) (B) and IL-10^+^ PMN-MDSCs (CD66abce^+^ CD33^+^ IL-10^+^) (C) in naive, LTBI, and ATB groups. (D) Images showing the differential distribution of IL-10^+^ PMN-MDSCs in different granuloma regions. (E and F) Estimated fractions of IL-10^+^ PMNs (CD66abce^+^ IL-10^+^) (E) and IL-10^+^ PMN-MDSCs (CD66abce^+^ CD33^+^ IL-10^+^) (F) in the necrotic core (NC), intermediate myeloid layer (IM), lymphocytic cuff (LC), active lung interstitium (ALI), and naive lung interstitium (NLI). (G) Multilabel confocal images of PMN-MDSCs expressing MMP-9 in naive, LTBI, and ATB lung sections. (H and I) Graphical representation of fractions of PMNs expressing MMP-9 (CD66abce^+^ MMP-9^+^) (H) and PMN-MDSCs expressing MMP-9 (CD66abce^+^ CD33^+^ MMP-9^+^) (I). (J) Images showing distribution of MMP-9^+^ PMN-MDSCs in different layers of TB granuloma. (K and L) Graphs depicting the spatial distribution of MMP-9^+^ PMNs (CD66abce^+^ MMP-9^+^) (K) and MMP-9^+^ PMN-MDSCs (CD66abce^+^ CD33^+^ MMP-9^+^) (L). Statistical significance by one-way ANOVA with Tukey’s multiple-testing correction: *, *P* < 0.05; **, *P* < 0.01; ***, *P* < 0.001; ****, *P* < 0.0001. Data represent mean ± SEM (*n* = 4).

**FIG 5:**
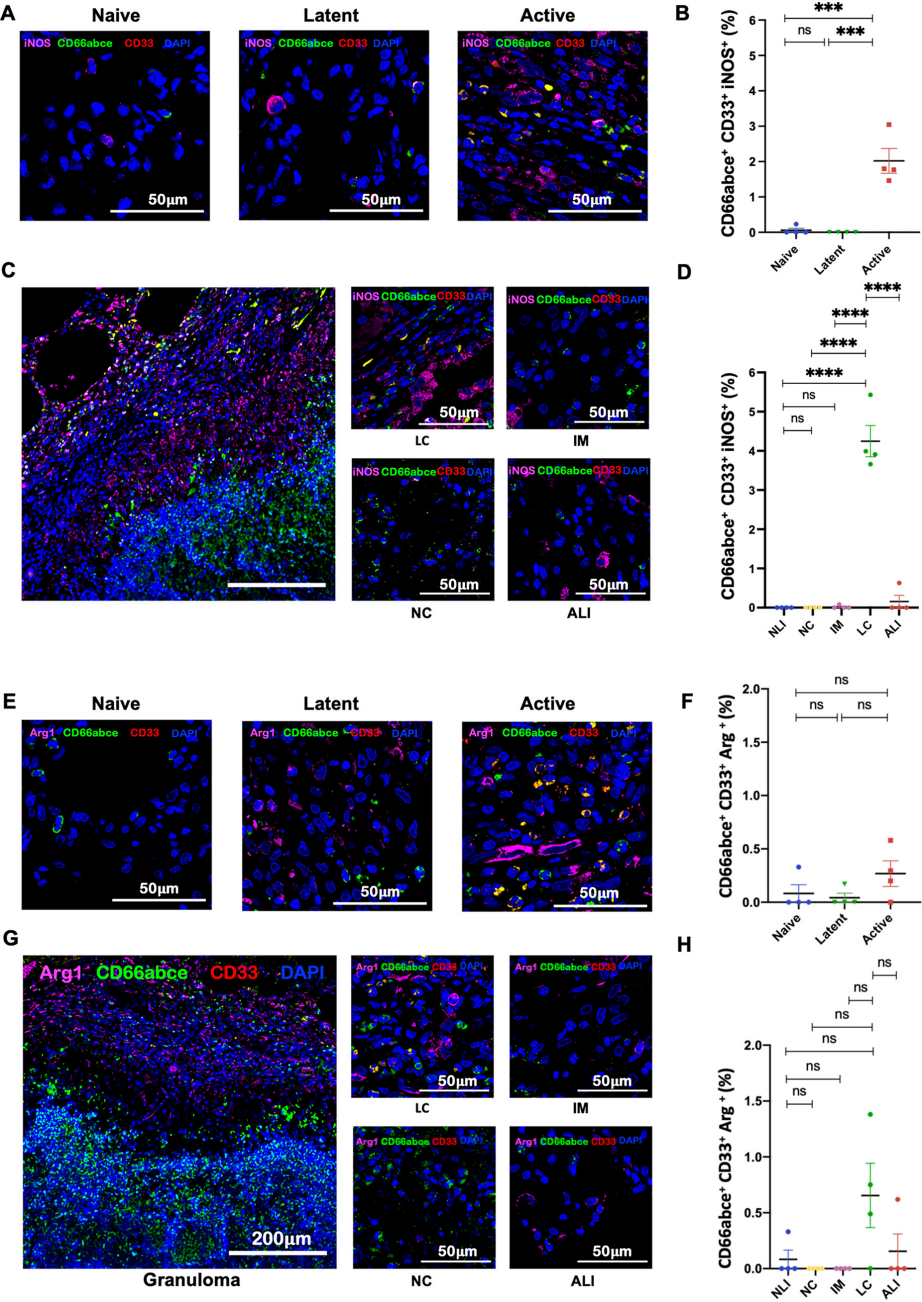
PMN-MDSCs in the lungs of M. tuberculosis-infected macaques express iNOS but not arginase. (A and E) Multilabel confocal images exhibiting the expression of iNOS (A) and arginase (E) by PMN-MDSCs in sections of naive, LTBI, and ATB lungs. (B and F) Graphical representation of the fractions of PMN-MDSCs expressing iNOS (CD66abce^+^ CD33^+^ iNOS^+^) (B) and arginase (CD66abce^+^ CD33^+^ Arg^+^) (F) in naive, LTBI, and ATB groups. Shown are the images depicting the distribution of iNOS^+^ PMN-MDSCs (C) and arginase^+^ PMN-MDSCs (G) in various layers of granuloma. (D and H) Estimated fractions revealing the distribution of PMN-MDSCs expressing iNOS (CD66abce^+^ CD33^+^ iNOS^+^) (D) and PMN-MDSCs expressing arginase (CD66abce^+^ CD33^+^ Arg^+^) (H) in the necrotic core (NC), intermediate myeloid layer (IM), lymphocytic cuff (LC), active lung interstitium (ALI), and naive lung interstitium (NLI). Statistical significance by one-way ANOVA with Tukey’s multiple-testing correction: *, *P* < 0.05; **, *P* < 0.01; ***, *P* < 0.001; ****, *P* < 0.0001. Data represent mean ± SEM (*n* = 4).

**FIG 6:**
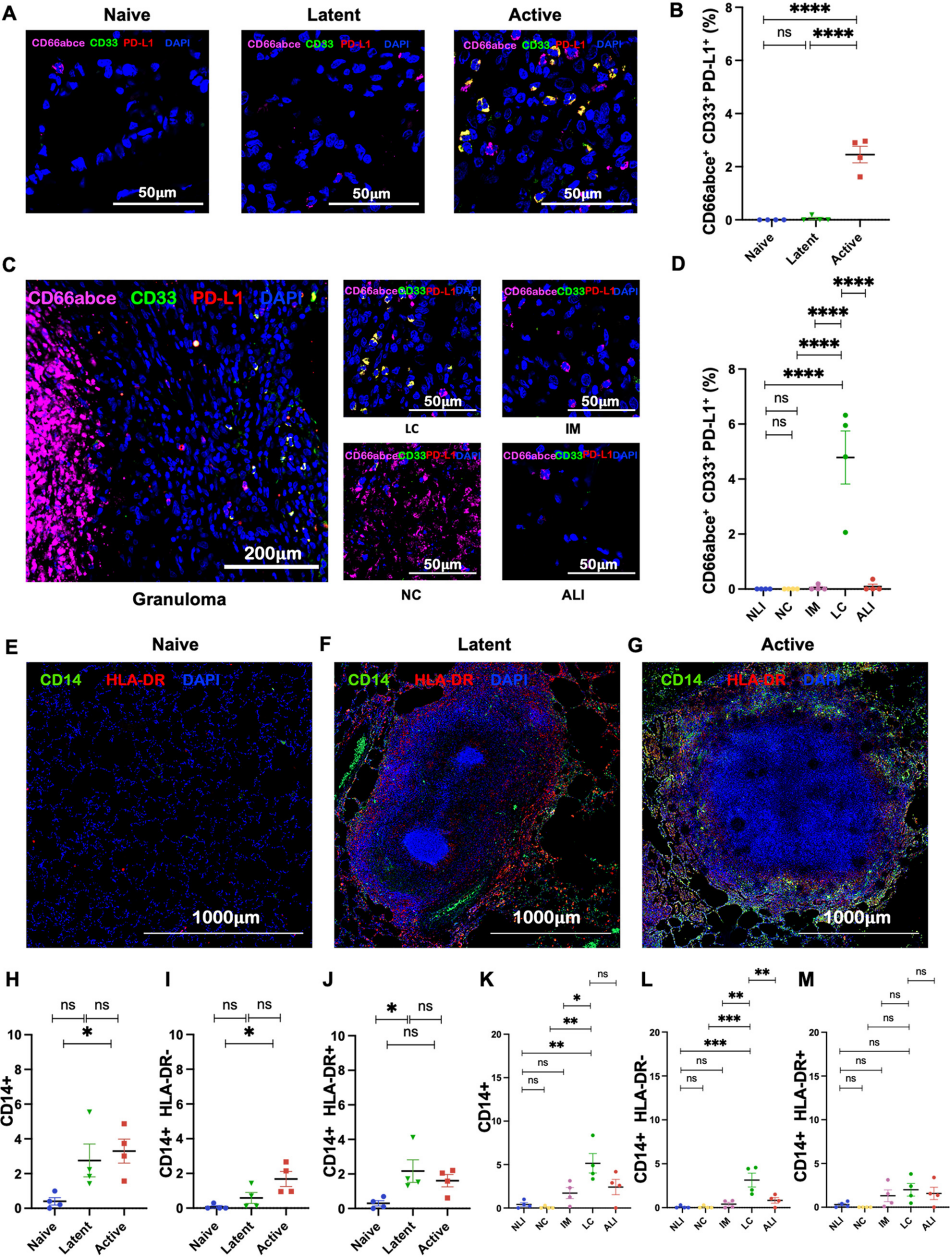
PMN-MDSCs in the lungs of M. tuberculosis-infected macaques express higher PD-L1. (A and C) Multilabel immunohistochemistry images depicting the expression of programmed cell death ligand-1 (PD-L1) by MDSCs in naive, LTBI, and ATB lung sections (A) and different regions of granuloma (C). (B and D) Graphical representation of the fractions of PD-L1^+^ PMN-MDSCs (CD66abce^+^ CD33^+^ PD-L1^+^) in naive, LTBI, and ATB groups (B), as well as in the necrotic core (NC), intermediate myeloid layer (IM), lymphocytic cuff (LC), active lung interstitium (ALI), and naive lung interstitium (NLI) (D). (E to G) Tiled confocal images (4-by-4 fields at ×10 magnification) from lung sections of naive (E), LTBI (F), and ATB (G) lung sections stained with CD14 (green) and HLA-DR (red), depicting the distribution of monocytic-myeloid-derived suppressor cells (M-MDSCs) represented by CD14^+^ HLA-DR^−^. (H to J) Estimated fractions of monocytes (CD14^+^) (H), M-MDSCs (CD14^+^ HLA-DR^−^) (I), and immunosuppressive monocytes (CD14^+^ HLA-DR^+^) (J) in naive, latent, and ATB groups. (K to M) Spatial distribution of monocytes (CD14^+^) (K), M-MDSCs (CD14^+^ HLA-DR^−^) (L), and immunosuppressive monocytes (CD14^+^ HLA-DR^+^) (M) in the NC, IM, LC, ALI, and NLI. Statistical significance by one-way ANOVA with Tukey’s multiple-testing correction: *, *P* < 0.05; **, *P* < 0.01; ***, *P* < 0.001; ****, *P* < 0.0001. Data represent mean ± SEM (*n* = 4).

During ATB, not only were MDSCs recruited to the lungs of RMs in greater numbers and localized to the lymphocytic cuff of granuloma, but they also exhibited higher Ki67 expression (Fig. 3A to G). This result suggests either a higher proliferative potential in MDSCs *in situ* or their recent arrival from the periphery (or both). RMs with ATB also harbored MDSCs with significantly higher expression of IL-10 (Fig. 4A to F), MMP-9 (Fig. 4G to L), iNOS (Fig. 5A to D), and PD-L1 (Fig. 6A to D). Expression of these markers of T cell dysfunction was highest in MDSCs located in the lymphocytic cuffs. We did not, however, observe any significant differences in arginase expression across groups or layers of granuloma (Fig. 5E to H), indicating that arginase is neither constitutively expressed nor a crucial immunosuppressive mediator for MDSC-driven immune dysfunction in the lungs of ATB macaques. Functional studies in murine tumor models have also reported and validated that arginase is not inherently expressed in MDSCs and is not required by MDSCs for eliciting an immunosuppressive function (54).

Although comparatively infrequent relative to PMN-MDSCs, the M-MDSC population in the lung followed the same trend in localization, with higher proportions being recruited in lungs of ATB animals (Fig. 6E to J) and localizing to the lymphocytic cuffs of granuloma (Fig. 6K to M).

## DISCUSSION

Localization of MDSCs to malignant tissues is accompanied by an increase in their frequencies in the peripheral circulation. MDSCs from both intratumoral regions and peripheral circulation induce similar immunosuppressive capabilities, and their recruitment has been suggestive of severity and progression of cancer as well as treatment failure (22, 55). A number of studies have established a similar increase in MDSC frequencies associated with ATB in murine models (10, 36) and human patients (7–9, 30, 33, 41). These studies also observed similar phenotypes and functions of MDSCs in murine (10, 36) and human (7–9, 30, 33, 41) ATB. Furthermore, MDSCs obtained from peripheral blood mononuclear cells (PBMCs), BAL fluid, and pleural effusions of ATB patients also demonstrated similar immunosuppressive capabilities, with a positive correlation with progression and inverse correlation with treatment success (7–9, 30, 33, 41). However, studies to interrogate phenotypic, functional, and localization aspects of MDSCs in the TB granulomas remain scarce, due to the challenges associated with sampling granulomas from human lungs and the inability of most murine models to mimic human-like granulomas.

We used the RM model of TB to overcome this knowledge gap and characterize MDSCs associated with lung TB granulomas. As expected, higher frequencies of MDSCs in blood and BAL fluid were associated with ATB compared to LTBI or baseline. Increased localization of PMN-MDSCs to the lungs of RMs with ATB was specifically observed. MDSC accumulation in lung parenchyma associated with progression of TB has been reported in murine models (45, 56). M. tuberculosis infection in necrosis-prone NOS2^−/−^ mice has attributed MDSC localization to the edges of necrotic granulomas (9, 56). NHPs in the ATB group also recorded a similar absence of MDSCs in the internal core or myeloid layers of granuloma. MDSCs specifically localized to the outer lymphocytic cuffs at the periphery of TB granulomas of ATB macaques. TB granulomas are known to have dysfunctional T cell responses and restricted T cell access (57–60). While this phenomenon was broadly believed to be induced by MDSCs, our findings support the premise that MDSCs in the NHP TB model restrict T cells and suppress their effector functions at the periphery of the granulomas. Despite PMNs being the abundant population in the granuloma core and macrophages primarily constituting the intermediate myeloid layer, MDSCs demonstrated a preferred localization to the lymphocytic cuff. Another trait of these MDSCs was higher Ki67 expression, which is indicative of recent differentiation from a proliferating precursor. The PMN populations from the core exhibit a lack of Ki67 expression indicative of longer survival time, possibly because of M. tuberculosis-induced inhibition of constitutive apoptotic pathways leading to delayed cell death (61, 62).

Proliferative signatures in MDSCs suggest their rapid turnover at this crucial immune synapse—the periphery of the granuloma. Furthermore, our results suggest downstream dysfunctional T cell responses against M. tuberculosis in animals with increased MDSC recruitment. Due to their unique localization to the lymphocytic cuff of the granuloma, PMN-MDSCs are important immunocytes regulating granuloma dynamics, and our results at least partially explain the restriction of T cell access and dysfunctional anti-TB activity in the active granuloma. The findings in this study suggest that adjunctive therapeutics targeting MDSCs could be a potential strategy for improving treatment outcome by helping to resolve the granuloma and consequently hasten clearance of M. tuberculosis within this unique environment.

Inflammation and inflammatory chemokines efficiently drive MDSC growth and recruitment. TB-induced inflammatory responses may drive the localized recruitment and systemic proliferation, as has been observed in cancer. MDSC-driven immunosuppression can also promote TB growth and trigger dysfunctional adaptive responses. While both of these effects appear to be convergent, our current data are based on the distinct modeling of different disease conditions (i.e., LTBI versus ATB versus naive macaques) and cannot quantify the *in vivo* immune scenario in a fully accurately manner. The comprehensive spatial analysis of granulomatous and nongranulomatous lung sections from naive, LTBI, and ATB animals helps clarify the role of MDSCs in potentially driving immunological dysfunction at the host-pathogen interface in granulomas by restricting T cell access to internal layers of the granuloma and by suppressing T cell function at the periphery. Further, interrogation of the underlying mechanisms needs an entirely new set of experiments involving functional blockade of MDSCs and/or MDSC mediators.

## MATERIALS AND METHODS

### Animals, infection, and treatment.

We randomly selected 6 TB-naive RMs and 6 RMs infected with a low dose of M. tuberculosis CDC1551 via aerosol, which maintained LTBI for 9 weeks postinfection, and 6 RMs infected with a high dose of M. tuberculosis CDC1551, again via aerosol, which developed ATB characterized by pyrexia, rapid weight loss, elevated serum CRP levels, high CXR scores, and detection of viable M. tuberculosis CFU in the BAL fluid and lungs. Two of the seven animals in the active TB group were female. However, female animals were not included in the other groups. The three groups of NHPs belong to different cohorts, but the NHPs with latent and active TB were from the same group of macaques infected with low and high doses of M. tuberculosis, respectively. Low-dose M. tuberculosis infection involved depositing 5 to 10 CFU of the M. tuberculosis CDC1551 strain into the lungs of RMs, while high-dose infection deposited 50 to 100 CFU of M. tuberculosis CDC1551. The animals were subjected to weekly physical examinations by veterinarians, including body temperature and weight, and complete blood chemistries, including CRP, were evaluated.

### Measurement of M. tuberculosis infection progression and TB disease.

To measure the extent of TB disease following aerosol M. tuberculosis infection in the various groups of animals, we studied serum CRP levels weekly, along with CXR and viable M. tuberculosis CFU recorded in lungs at necropsy, as described earlier (59, 63–68).

### Flow cytometry.

Flow cytometry was performed on whole blood and lung samples from all animals, as previously described (59, 63–68). At necropsy, PBMCs and lung cells were isolated from each animal for flow cytometric analysis to characterize MDSCs. Antibodies used for analysis of MDSC populations included the following: from BD Biosciences, CD3 (clone SP34-2), CD4 (clone L200), CD8 (clone RPA-T8), CD20 (clone 2H7), HLA-DR (clone L243), anti-CD11b (ICRF44), anti-CD14 (M5E5), and Ki67 (clone B56), and from Miltenyi Biotec (Auburn, CA), anti-CD33 (clone AC104.3E3) and anti-CD66abce (TET2) (59, 63).

### Confocal microscopy.

Multilabel fluorescence immunohistochemistry was performed on formalin-fixed, paraffin-embedded tissue as described previously (69, 70). Briefly, formalin-fixed lung sections were cut into 5-μm-thick sections. Slides were deparaffinized in xylenes and underwent rehydration with subsequent gradations of ethanol and distilled water (dH_2_O). Heat-induced antigen retrieval (HIER) was performed using citrate buffer (10 mM [pH 6]) at 95 to 100°C for 20 min, followed by blocking in 3% bovine serum albumin (BSA)–Tris-buffered saline with Tween 20 (TBST). Subsequently, the slides were incubated with the respective primary antibodies for 2 h followed by subsequent incubation with the fluorophore-conjugated secondary antibody for 45 min at room temperature (RT). Slides were stained with DAPI (4′,6-diamidino-2-phenlyindole) and visualized using a Zeiss LSM 800 confocal microscope. Fifteen fields/animal/group were captured, and quantification was done using Image J software.

### Statistical analyses.

Statistical comparisons were performed using two-tailed unpaired *t* tests for comparisons involving two groups, whereas, one-way analysis of variance (ANOVA) in GraphPad Prism with Tukey’s correction for multiple comparisons was used whenever data sets involved more than two groups.

### Study approval.

All animal procedures and work were specifically approved by the Texas Biomedical Research Institute and Tulane National Primate Research Center Institutional Animal Care and Use Committees. All work related to biological containment was approved by the Texas Biomedical Research Institute and the Tulane Institutional Biosafety Committees.

10.1128/mbio.03189-21.3 TABLE S1 Age, gender, and MAMU-type of each Indian rhesus macaque included in this study. Download Table S1, XLSX file, 0.01 MB. Copyright © 2021 Singh et al. 2021 Singh et al. https://creativecommons.org/licenses/by/4.0/ This content is distributed under the terms of the Creative Commons Attribution 4.0 International license.

10.1128/mbio.03189-21.4 TABLE S2 Markers used for MDSC characterization by flow cytometry and immunohistochemistry (IHC). Download Table S2, XLSX file, 0.01 MB. Copyright © 2021 Singh et al. 2021 Singh et al. https://creativecommons.org/licenses/by/4.0/ This content is distributed under the terms of the Creative Commons Attribution 4.0 International license.
