# Supplementary figures and images for: Myeloid-Derived Suppressor Cells Mediate T Cell Dysfunction in Nonhuman Primate TB Granulomas
Source: mBio. 2021 Dec 14;12(6):e03189-21. doi: 10.1128/mbio.03189-21 (PMC8669465; doi:10.1128/mbio.03189-21)

Figure S1

A

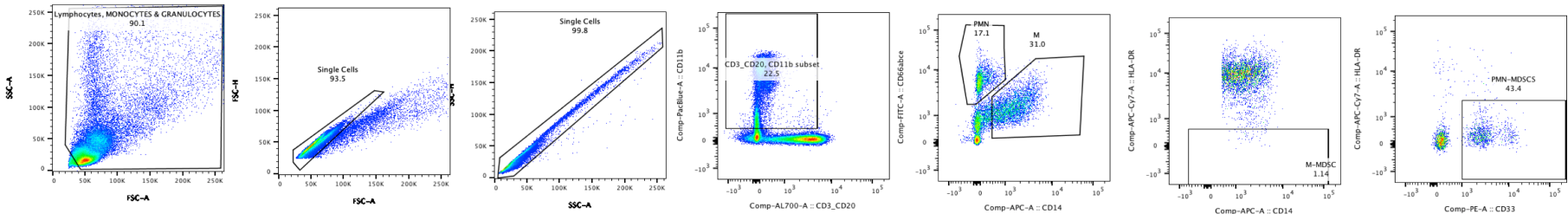

B

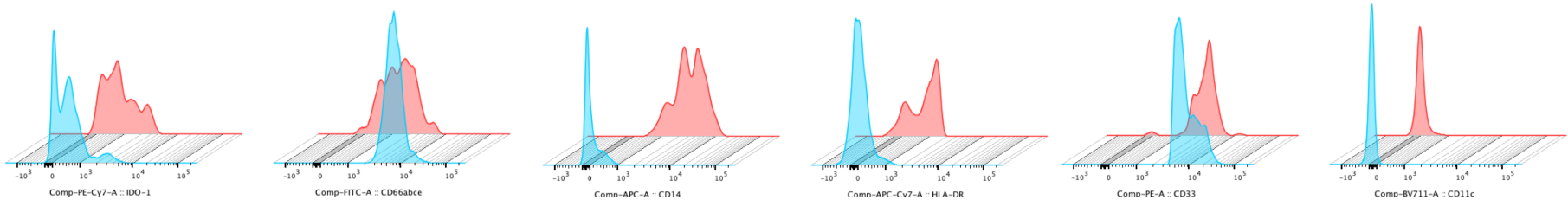

Supplement: FIG S1 [file mbio.03189-21-sf001.pdf]

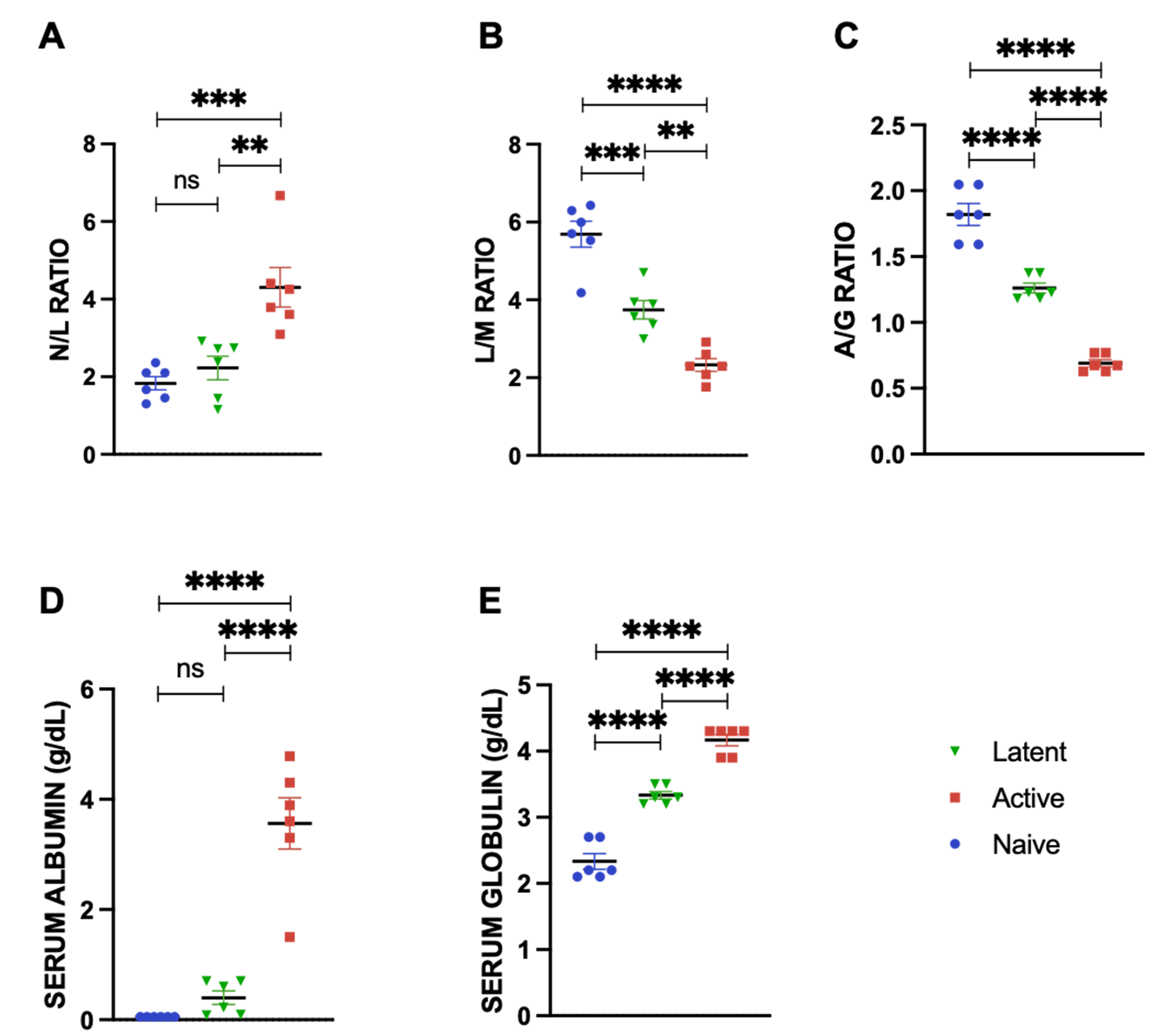

Supplement: FIG S2 [file mbio.03189-21-sf002.tiff]
